# *Fusobacterium nucleatum* elicits subspecies-specific responses in human neutrophils

**DOI:** 10.3389/fcimb.2024.1449539

**Published:** 2024-10-10

**Authors:** Maria Muchova, Sarah A. Kuehne, Melissa M. Grant, Peter P. Smith, Malee Nagi, Iain L. C. Chapple, Josefine Hirschfeld

**Affiliations:** ^1^ Periodontal Research Group, Birmingham School of Dentistry, Institute of Clinical Sciences, The University of Birmingham, Birmingham, United Kingdom; ^2^ School of Science and Technology, Nottingham Trent University, Nottingham, United Kingdom; ^3^ Birmingham Dental Hospital, Birmingham Community Health National Health Service (NHS) Foundation Trust, Birmingham, United Kingdom; ^4^ Birmingham National Institute for Health and Care Research (NIHR) Biomedical Research Centre (BRC) in Inflammation, Birmingham University, Birmingham, United Kingdom

**Keywords:** *Fusobacterium nucleatum*, subspecies, neutrophil, biofilm, immunogenicity

## Abstract

*Fusobacterium nucleatum* as a Gram-negative anaerobe plays a key bridging role in oral biofilms. It is involved in periodontal and extraoral diseases, the most prominent being colorectal cancer. Five subspecies are recognised: *animalis, fusiforme, nucleatum, polymorphum* and *vincentii*. Subspecies interact with neutrophils constantly patrolling tissues to remove microbial intruders. Neutrophil antimicrobial activities include generation of reactive oxygen species (ROS), formation of neutrophil extracellular traps (NETs) and release of cytokines and neutrophil enzymes. Subspecies-specific differences in immunogenicity have previously been observed in a neutrophil-like cell line but were not investigated in human neutrophils. Additionally, neutrophil responses to planktonic and biofilm-grown *F. nucleatum* have not been studied to date. The aims of this study were to compare the immunogenicity of planktonic and biofilm-grown *F. nucleatum* and to investigate potential differences in human neutrophil responses when stimulated with individual *F. nucleatum* subspecies. Human neutrophils isolated from peripheral blood were stimulated with planktonic and biofilm-grown *F. nucleatum* subspecies. Generation of ROS and NET formation were quantified by luminescence and fluorescence assays, respectively. Secretion of cytokines (IL-1β, TNF-α, IL-6, IL-8), neutrophil elastase and matrix metalloproteinase-9 was quantified by enzyme-linked immunosorbent assay (ELISA). Neutrophil responses showed biofilm-grown bacteria induced a significantly higher total and intracellular ROS response, as well as shorter time to total ROS release. Biofilm-grown *F. nucleatum* led to significantly lower IL-1β release. We found significant differences among individual subspecies in terms of total, intracellular ROS and extracellular superoxide. Subspecies *polymorphum* stimulated the highest mean amount of NET release. Amounts of cytokines released differed significantly among subspecies, while no differences were found in lysosomal enzyme release. Immunogenicity of *F. nucleatum* in human neutrophils is highly subspecies-specific *in vitro* with regard to ROS release and cytokine production. Understanding subspecies-specific immunogenicity of *F. nucleatum* may facilitate the discovery of novel therapeutic targets in *F. nucleatum*-mediated diseases.

## 1 Introduction

*Fusobacterium nucleatum* is a Gram-negative anaerobe present in the healthy oral microbiome (Han, 2015), where it plays a key bridging role between early, beneficial colonisers in oral biofilms, such as *Streptococcus* species, and late, pathogenic colonisers, such as *Porphyromonas gingivalis* (Jung et al., 2017). *F. nucleatum* is considered an opportunistic pathogen due to its involvement in periodontal disease as well as in extraoral diseases, like cardiovascular disease, inflammatory bowel disease, various types of cancers (Fan et al., 2023) and likely endometriosis (Muraoka et al., 2023). At present, five subspecies are recognised: *animalis*, *fusiforme*, *nucleatum*, *polymorphum* and *vincentii* (Han, 2015). Some authors, however, class *fusiforme* and *vincentii* under one subspecies *vincentii* (Kook et al., 2013).

Whether in the oral cavity or in extraoral tissues, *F. nucleatum* subspecies interact with leukocytes responding to the presence of bacteria, with neutrophils among the first responders as part of the innate immune system. Also known as neutrophilic polymorphonuclear leukocytes, neutrophils are the most numerous immune cell in human blood (Rosales, 2018). Their antimicrobial activities include generation of intra- and extracellular reactive oxygen species (ROS) (Nguyen et al., 2017), such as the superoxide anion and hydrogen peroxide. Microorganisms are also cleared following the release of DNA in the form of neutrophil extracellular traps (NETs) (Hirschfeld et al., 2015) and secretion of proteolytic enzymes including human neutrophil elastase (HNE) in the process of degranulation (Zeng et al., 2023). Neutrophils also release matrix metalloproteinase 9 (MMP-9), which degrades components of the extracellular matrix and plays a role in neutrophil transit through tissues and tissue remodelling (Wang, 2018; Velasquez et al., 2023).

Apart from the direct use of their antimicrobial arsenal, neutrophils also orchestrate the inflammatory response and involvement of other immune cells by releasing cytokines, such as TNF-α, IL-1β, IL-6 and IL-8 (Ling et al., 2015). Despite killing microbial invaders, neutrophils may also cause collateral tissue damage, especially if their functional response is abnormal (Filep, 2022). Neutrophils from patients with periodontitis were found to be hyper-reactive upon stimulation with *F. nucleatum* ssp. *polymorphum*, generating significantly higher amounts of ROS compared to healthy controls (Matthews et al., 2007). Periodontitis-related neutrophil hyper-reactivity was also detected in terms of significantly higher cytokine release when *F. nucleatum* ssp. *polymorphum* was used, among other stimuli (Ling et al., 2015).

In the context of colorectal cancer (CRC), Kong et al. (2023) detected higher neutrophil infiltration in CRC tissues compared to adjacent tissues. Moreover, *F. nucleatum*-infected CRC tissues contained significantly higher numbers of neutrophils as well as significantly greater amounts of NETs. Further *in vitro* investigations using *F. nucleatum* ssp. *nucleatum* showed that *F. nucleatum*-induced NETs stimulated angiopoiesis and tumour metastasis (Kong et al., 2023).

Although much research is currently being conducted investigating the *F. nucleatum*-related damage caused by neutrophils in various diseases, there is a paucity of data comparing neutrophil responses to all *F. nucleatum* subspecies. It is imperative that more research is performed due to differential involvement of individual subspecies in healthy and diseased conditions: ssp. *fusiforme* and *vincentii* were isolated from healthy sites (Gharbia et al., 1990; Han, 2015), while ssp. *animalis* and *nucleatum* have been related to disease (Gharbia et al., 1990; Gmür et al., 2006; Feres et al., 2018). Ssp. *polymorphum* has been found to be associated with both healthy sites and diseased lesions (Feres et al., 2018). Subspecies-specific differences in immunogenicity were observed in the HL-60 neutrophil-like cell line (Kurgan et al., 2017), but, to the best of our knowledge, this was not investigated in primary human neutrophils. Additionally, most studies employ planktonic bacterial cultures for neutrophil stimulation. However, considering that the expression of virulence genes in biofilms is significantly higher than in planktonic cultures (Becker et al., 2001; Resch et al., 2005) and *F. nucleatum*-containing oral biofilms are crucial in development of periodontal disease (Vieira Colombo et al., 2016), utilising biofilm stimuli in *F. nucleatum* pathogenicity studies might provide more clinically relevant results.

Therefore, the aims of this study were to compare the immunogenicity of planktonic and biofilm-grown *F. nucleatum* and to investigate potential differences in human neutrophil responses when stimulated with individual *F. nucleatum* subspecies. We hypothesised that there is a difference in neutrophil responses to planktonic and biofilm-grown *F. nucleatum*, and that there is a difference among subspecies in their ability to stimulate ROS generation, NET formation, pro-inflammatory cytokine and neutrophil enzyme release. We successfully identified differences between planktonic and biofilm *F. nucleatum* stimuli, and we determined distinct subspecies-specific stimulation of functional and molecular responses of human neutrophils.

## 2 Materials and methods

### 2.1 Bacterial strains and culture conditions

*F. nucleatum* type strains, originally obtained from the American Tissue Culture Collection (ATCC) and maintained in the Periodontal Research Group culture collection (School of Dentistry, University of Birmingham, UK) were used in this study: *F. nucleatum* ssp. *animalis* ATCC 51191 (FNA), *F. nucleatum* ssp. *fusiforme* ATCC 51190 (FNF), *F. nucleatum* ssp. *nucleatum* ATCC 25586 (FN25), *F. nucleatum* ssp. *polymorphum* ATCC 10593 (FNP), and *F. nucleatum* ssp. *vincentii* ATCC 49256 (FNV). A genetically tractable strain *F. nucleatum* ssp. *nucleatum* ATCC 23726 (FN23), donated by Dr Daniel Slade (Virginia Polytechnic Institute and State University, Blacksburg VA, USA), was also used in the study. This strain allows utilisation of a well characterised and optimised gene deletion system (Casasanta et al., 2020) and available mutants are of this background. Thus, wild-type FN23 strain was used in order to compare the immunogenicity of the reliably genetically modifiable parental strain with the remaining subspecies and to establish a knowledge base for potential future experiments utilising modified FN23.

All subspecies were grown on Schaedler anaerobe agar plates (SAA; Sigma-Aldrich/Merck, Darmstadt, Germany) at 37°C in an anaerobic chamber (80% N_2_, 10% CO_2_, and 10% H_2_; Don Whitley DG250 Anaerobic Workstation, Don Whitley Scientific, Bingley, UK). Planktonic cultures were grown anaerobically and stationary in Schaedler anaerobe broth (SAB; Oxoid, Basingstoke, UK).

### 2.2 Stimuli for neutrophils

Human neutrophils in this study were stimulated with live planktonic (subspecies with prefix “p”) and biofilm-grown (subspecies with prefix “b”) *F. nucleatum*. Planktonic stimuli were prepared as follows: SAB was inoculated with each subspecies and incubated for 18 hours anaerobically. Cultures were washed once with phosphate buffered saline (PBS), then adjusted to OD_600 _= 1 in PBS. Each adjusted subspecies was mixed with glycerol (15% v/v final concentration) and aliquots were stored at -80°C. Storage was confirmed not to affect bacterial viability by carrying out quantification of bacteria by colony counting.

*F. nucleatum* single-species biofilms were grown as described previously (Muchova et al., 2022) with minor modifications related to the bacterial volumes in the multi-well plates. Planktonic cultures were washed once with PBS, then adjusted to OD_600_ = 1 in SAB. 3 ml of the adjusted culture was added to wells of 6-well polystyrene plates (Nunclon™ Delta Surface, Thermo Scientific, Loughborough, UK) without additional surface coating and biofilms were grown for 72 hours anaerobically. Growth medium was changed after 24 and 48 hours of incubation and contamination monitoring was performed daily. After 72 hours of incubation, supernatant was carefully removed and single-subspecies biofilms were resuspended by vigorous pipetting in PBS in order to aid bacterial quantification and standardisation for the assays, while retaining the extracellular matrix biofilm components. Bacterial concentration was adjusted to OD_600_ = 1, subspecies were then mixed with glycerol (15% v/v final concentration) and aliquots stored at -80°C.

Bacterial numbers were determined for both planktonic and biofilm-grown bacteria by correlating OD to colony counts (CFU/ml) and the numbers of bacteria from glycerol stocks were standardised by further dilution in PBS for the assays. The final glycerol concentration after dilutions ranged from 2% to 0.5% and it was previously established that the remaining glycerol concentration did not affect neutrophil function. Incubation of neutrophils with bacteria was performed in the culture medium specific for each assay.

### 2.3 Isolation of neutrophils from peripheral blood

Peripheral blood was collected from healthy adult volunteers after obtaining informed consent (Ethics reference BCHCDent024.2024) in lithium heparin vacutainers (Greiner Bio-One, Austria) by venepuncture. Neutrophils were isolated as detailed in Matthews et al. (2007) using discontinuous Percoll density gradients (1.079 g/ml on top of 1.098g/ml gradient; Cytiva, USA). Erythrocytes were removed by incubation in lysis buffer [detailed composition in Matthews et al. (2007)]. Cells were resuspended in PBS, counted using trypan blue dye, and the cell number was adjusted in the corresponding medium used in assays.

### 2.4 Enhanced chemiluminescent quantification of reactive oxygen species

ROS quantification was performed as previously described (Matthews et al., 2007), with the modifications detailed below. Neutrophils were adjusted to 1 x 10^6^ cells/ml in glucose supplemented PBS (GPBS: Dulbecco’s phosphate buffered saline no calcium, no magnesium, Gibco™, Thermo Scientific, Loughborough; 10mM glucose; 1.35 mM CaCl_2_; 1.5 mM MgCl_2_) and 10^5^ cells/well were added to a white 96-well plate (Sterilin™ White Microtiter™ Plates, Thermo Fisher Scientific, Loughborough, UK) pre-blocked with 1% w/v bovine serum albumin (BSA) in PBS overnight at 4°C. To quantify total ROS (intracellular and extracellular), 30 μl of luminol (3 mmol/l, pH 7.3; Sigma-Aldrich/Merck, Darmstadt, Germany) with horseradish peroxidase (HRP, Type XII, 7.5 U/ml in the well; Sigma-Aldrich/Merck, Darmstadt, Germany) was used. To quantify intracellular ROS, 30 μl of luminol was combined with superoxide dismutase (SOD, bovine, 50 U/ml in the well; Sigma-Aldrich/Merck, Darmstadt, Germany), catalase (from bovine liver, 20 U/ml in the well; Sigma-Aldrich/Merck, Darmstadt, Germany) and horseradish peroxidase (7.5 U/ml). Lucigenin (30 μl, 0.33 mg/ml) was used to measure superoxide generation.

Plates were placed into the luminometer (Infinite 200 PRO, Tecan, Switzerland) and equilibrated at 37°C for 30 minutes. Cells were then stimulated with *F. nucleatum* subspecies (multiplicity of infection (MOI) 100) and light output was measured for a further 150 minutes. Results were expressed as peak relative light units (RLU). Assays were performed in technical duplicate, with N=8 healthy adult volunteers for total and intracellular ROS quantification, and N=6 healthy adult volunteers for superoxide generation.

### 2.5 Quantification and fluorescent microscopy of neutrophil extracellular traps

NETs were quantified as described by Palmer et al. (2012) with modifications (described next). Isolated neutrophils [1 x 10^5^ cells in RPMI 1640 medium (without L-glutamine and phenol red; Cytiva, USA)] were added to a pre-blocked 96-well plate (1% w/v BSA in PBS). Following equilibration, neutrophils were stimulated with *F. nucleatum* subspecies (MOI 100:1). Cells were further incubated for 120 minutes.

To confirm NET release, neutrophils were fixed with 2% paraformaldehyde for 10 minutes at room temperature and stained with SYTOX green (diluted in PBS, concentration in the well 1.25 µM, excitation/emission 485/535 nm; Invitrogen, USA) for 10 minutes in the dark. Neutrophils were gently washed with PBS twice and visualised using an epifluorescence microscope at 20X magnification (Nikon Eclipse TE300 with a coolLED PE-100 LED excitation system).

To quantify NETs, micrococcal nuclease (15 µl, 1U/ml, Worthington, USA) was added after 120 minutes to digest extruded DNA for 10 minutes at 37°C. The microplate was centrifuged and the supernatant mixed with SYTOX green (concentration in the well 1 µM; Invitrogen, USA) diluted in PBS in a black 96-well plate (Thermo Scientific™ Sterilin™ Microtiter™ Plates, Thermo Fisher Scientific, Loughborough, UK). DNA fluorescence was read using a microplate reader (Spark^®^, Tecan; excitation 485 nm, emission 525 nm). Readings were expressed as relative fluorescence units (RFU). Bacterial fluorescence was quantified separately and subtracted from the neutrophil readings. Quantification was performed in technical duplicate, with N=8 healthy adult volunteers for RPMI, and N=10 healthy adult volunteers for test samples.

### 2.6 Quantification of cytokine and neutrophil enzyme release by enzyme-linked immunosorbent assay

Isolated neutrophils were incubated as described previously (Ling et al., 2015). Cells were diluted to 2.5 x 10^6^ cells/ml in supplemented RPMI (sRPMI: RPMI 1640, without L-glutamine and phenol red, Cytiva, USA; 10% heat-inactivated FBS; 25 mM HEPES, Sigma-Aldrich/Merck, Darmstadt, Germany; 2 mM glutamine). Neutrophils were then stimulated with *F. nucleatum* subspecies diluted in sRPMI (MOI 100:1) and incubated at 37°C, 5% CO_2_ for up to 18 hours: culture supernatants were collected either after 18 hours for endpoint quantification or after 1, 2, 4, 6 and 18 hours of incubation for time-course quantification. Commercial ELISA kits (DuoSet^®^, R&D Systems, Abingdon, UK) were used to quantify human cytokines IL-1β, IL-6, TNF-α, IL-8 and neutrophil enzymes matrix metalloproteinase 9 (MMP-9) and human neutrophil elastase (HNE/ELA2). All ELISA measurements were performed in technical duplicates and according to the manufacturer’s instructions. The lowest concentration of each standard was chosen as the limit of quantitation (LOQ). Assays were performed in technical duplicate, for 5 healthy adult volunteers for endpoint quantification, and 3 healthy adult volunteers for time-course quantification.

### 2.7 Quantification and light microscopy of necrosis

Viability of neutrophils stimulated with *F. nucleatum* subspecies was assessed by measuring necrosis following the manufacturer’s instructions (RealTime-Glo™ Annexin V Apoptosis and Necrosis Assay, Promega, UK). Briefly, isolated neutrophils diluted in sRPMI (1 x 10^5^ cells in 100 µl) were stimulated either with a positive control (50 µl; 50 µg/ml LL-37) or with *F. nucleatum* subspecies (MOI 100:1; 50 µl). 100 µl of 2X detection reagent was added, the cells were incubated for 18 h at 37°C (Infinite 200 PRO, Tecan, Switzerland) and fluorescence was recorded automatically every 15 minutes. Assays were performed in technical duplicate with healthy adult volunteers (N=4). Morphological changes of necrotic neutrophils were confirmed by light microscopy after 330 minutes of incubation and images were taken at 60X magnification (Nikon Eclipse TE300).

### 2.8 Statistical analysis

GraphPad Prism (version 10.1.1 for Windows, Boston, Massachusetts USA) was used to perform statistical analysis and to generate graphs. Normality of the datasets was tested using the Shapiro-Wilk test. If the data were normally distributed, one-way ANOVA with Tukey *post hoc* test was performed. If the data were not normally distributed, either Mann-Whitney U or Krustal-Wallis tests followed by Dunn’s *post hoc* test were utilised. Statistical tests used in each assay are specified in the figure legends. Statistical significance was considered if p < 0.05.

## 3 Results

### 3.1 Planktonic and biofilm-grown *F. nucleatum* stimulate differential responses in human neutrophils

**Figure 1:**
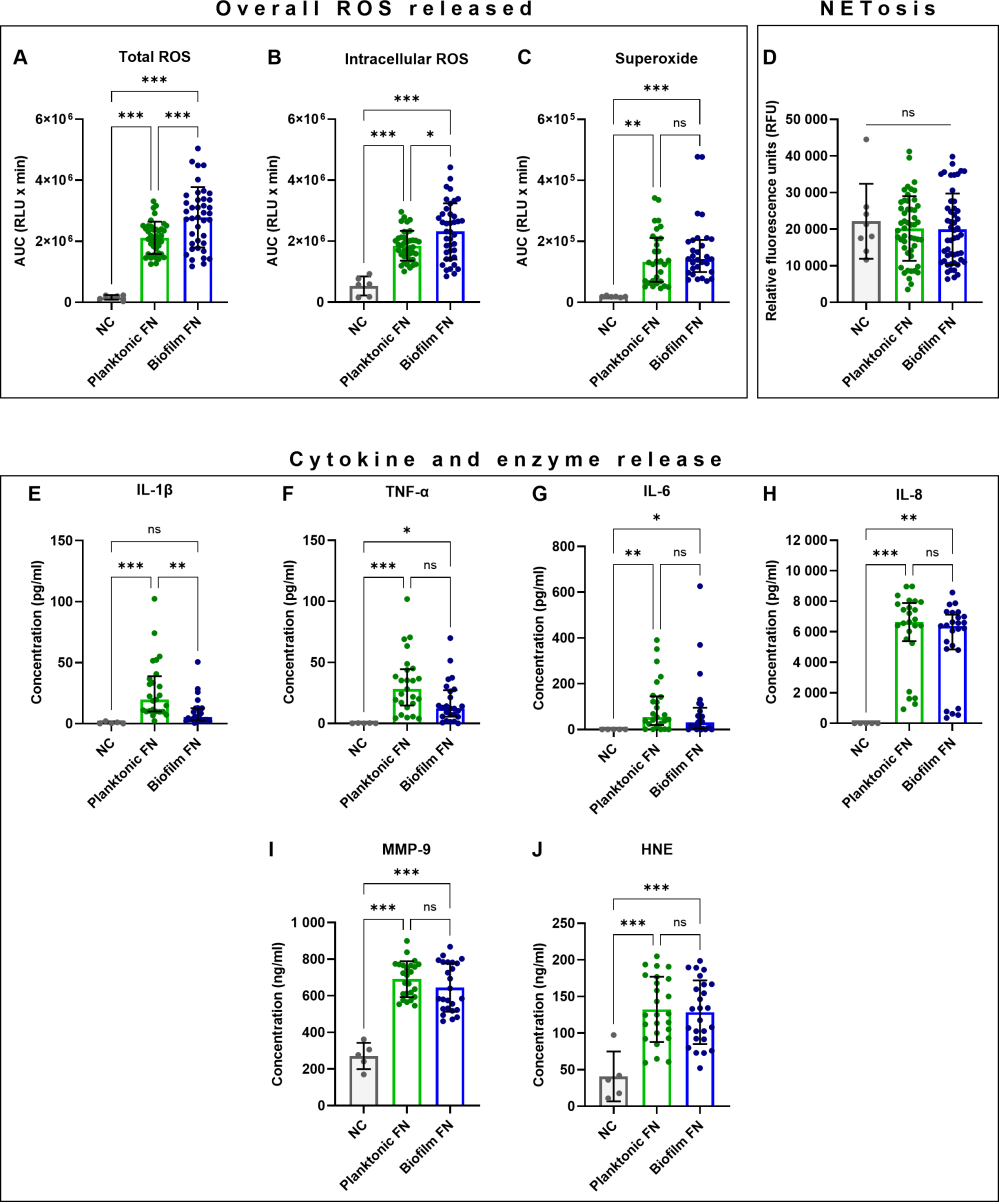
Comparisons of planktonic (p) and biofilm-grown (b) *F*. *nucleatum* (FN) stimulating antimicrobial responses in human neutrophils. **(A–C)** Quantification of overall ROS released shown as area under the curve (AUC). **(D)** Quantification of NET-DNA. **(E–H)** Quantification of cytokine and **(I–J)** antimicrobial enzyme release. Numbers of samples in each data set analysed are specified in **Supplementary Table 1**. ns, not significant; *p<0.05; **p<0.01; ***p<0.001. **(A, B, D, I, J)** One-Way ANOVA with Tukey’s *post hoc* test, mean values and SD are shown. **(C, E–H)** Kruskal-Wallis test with Dunn’s *post hoc* test, values shown as median and interquartile range (IQR).

To test the hypothesis that biofilm-grown *F. nucleatum* generated differential biological neutrophil response from their planktonic counterparts, an *a priori* decision was made to combine data points from neutrophil stimulations following exposure to individual *F. nucleatum* subspecies grown planktonically and in single-subspecies biofilms into two groups: “planktonic” versus “biofilm-grown”. This approach allowed analysis at a species level, combining all subspecies except FNP due to its inability to grow biofilms *in vitro* (Muchova et al., 2022). Viability of live *F. nucleatum* stimuli, which were used for the assays, was not affected by storage conditions. The majority of analysed neutrophil responses did not differ between planktonic and biofilm-grown bacteria (**Figures 1C, D, F–J**). However, biofilm-grown bacteria induced a higher ROS response. Specifically, biofilm-grown *F. nucleatum* stimulated significantly higher total and intracellular peak ROS release (**Supplementary Table 1**) as well as total and intracellular overall ROS release (**Figures 1A, B**). Additionally, biofilm-grown *F. nucleatum* elicited a significantly faster neutrophil response when time to total peak ROS was measured (**Supplementary Table 1**). By contrast, biofilm-grown *F. nucleatum* led to significantly lower IL-1β release (**Figure 1E**).

It is noteworthy that a high degree of variation was observed in neutrophil responses (**Figure 1**; **Supplementary Table 1**), suggesting that subspecies-specific differences exist and are likely masked within these combined datasets. Therefore, detailed analyses of neutrophil responses to individual *F. nucleatum* subspecies were performed next.

### 3.2 Generation of ROS by human neutrophils in response to *F. nucleatum* is subspecies-specific

Generation of total ROS, intracellular ROS and superoxide by neutrophils in response to individual *F. nucleatum* subspecies grown planktonically and in biofilms was analysed by chemiluminescent assay. Analysis of ROS release was performed from different perspectives to provide more biologically relevant outcomes: peak ROS release was reported as an indicator of the magnitude of the response, time measured to peak ROS release was analysed in order to describe the speed of the response and overall ROS release indicated the antimicrobial and tissue destructive potential of the response.

**Figure 2:**
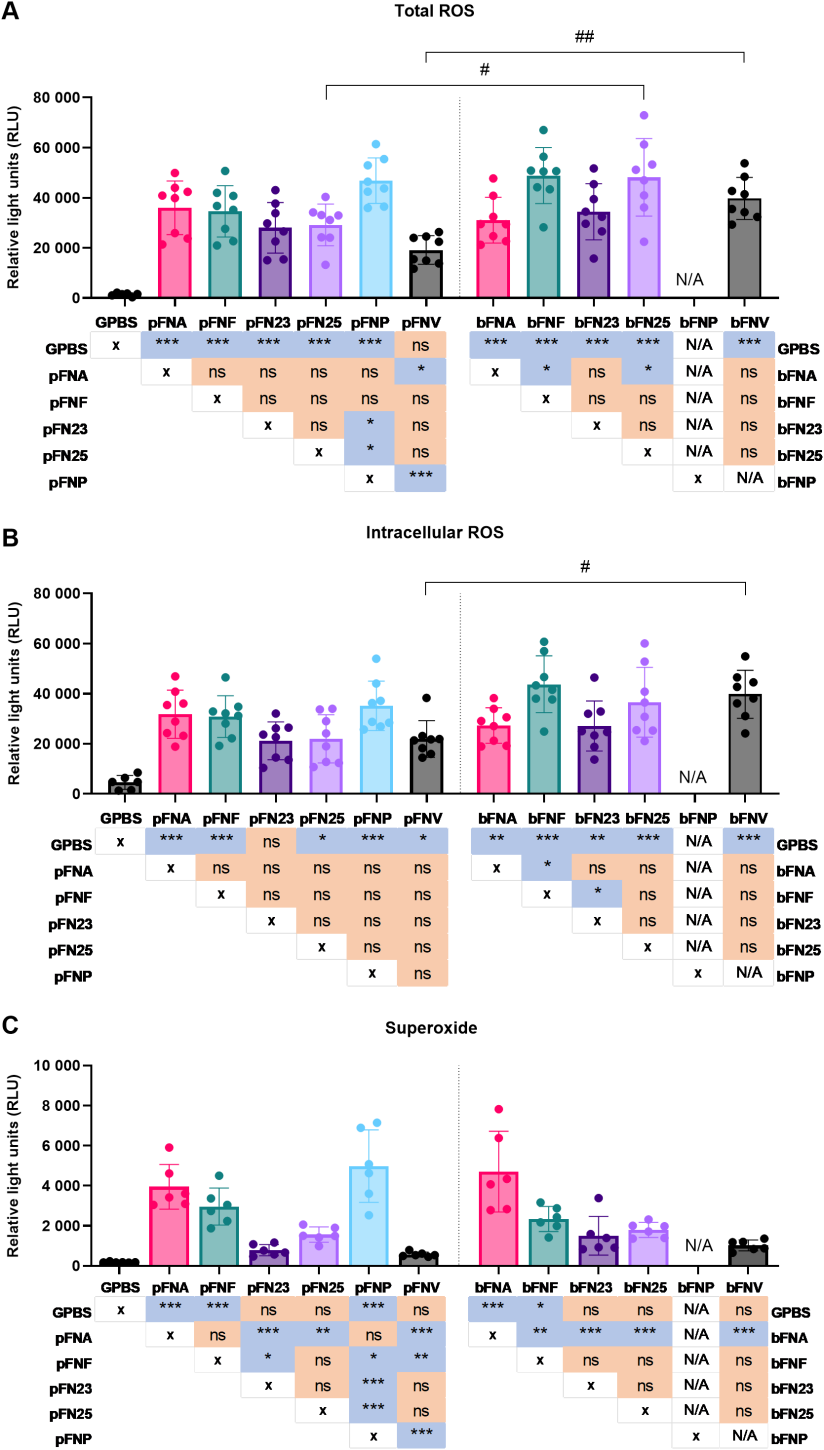
Quantification of peak ROS generation by neutrophils upon stimulation with *F. nucleatum* subspecies. Prefix “p” denotes planktonic subspecies, prefix “b” denotes biofilm-grown subspecies. Biofilm-grown FNP (bFNP) was unavailable due to the absence of biofilm formation. **(A)** Quantification of total ROS generation. All subspecies except pFNV are significantly higher than the negative control (GPBS), statistical significance detailed in a p-value matrix using “* (asterisk)” symbols. Significant differences between planktonic and biofilm-grown subspecies are indicated by “# (hash)” symbols. N=8 healthy adult volunteers. **(B)** Quantification of intracellular ROS generation. All subspecies except pFN23 are significantly higher than the negative control (GPBS), statistical significance detailed in a p-value matrix using “* (asterisk)” symbols. Significant difference between planktonic and biofilm-grown subspecies is indicated by “# (hash)” symbol. N=8 healthy adult volunteers. **(C)** Quantification of superoxide generation. N=6 healthy adult volunteers. Statistical significance detailed in a p-value matrix using “(asterisk)” symbols. All data are shown as mean values ±SD. Datasets were analysed using one-way ANOVA with Tukey’s post hoc test. ns – not significant; */# - p<0.05; **/## - p<0.01; *** - p<0.001.

Firstly, significant differences were identified among planktonic as well as biofilm-grown subspecies in total ROS generation, which comprises both intracellular and extracellular ROS. The significantly highest peak ROS release was stimulated by pFNP, bFNF and bFN25 (**Figure 2A**). Furthermore, bFN25 and bFNV elicited significantly higher peak ROS when compared to their planktonic counterparts. The fastest response was evoked by pFNP, while pFNV led to the slowest response on average (**Supplementary Figure 1A**). The highest overall total ROS generation was stimulated by bFN25 and bFNV and the amount of ROS generated was significantly higher when compared to their planktonic counterparts (**Supplementary Figure 2A**).

Secondly, in case of intracellular ROS, only bFNF led to significantly higher neutrophilic ROS generation compared to other subspecies, and the only difference between planktonic and biofilm stimuli was seen in FNV (**Figure 2B**). The fastest and slowest responses were elicited by pFNP and pFN25, respectively (**Supplementary Figure 1B**). Statistically significant differences were found among the biofilm-grown subspecies in terms of overall ROS release, and bFN25 and bFNV triggered significantly higher ROS generation compared to the planktonic stimuli (**Supplementary Figure 2B**).

Differences of a higher magnitude were found in superoxide generation. Planktonic subspecies pFNA and pFNP as well as bFNA elicited significantly higher superoxide generation when compared to the remaining subspecies, while planktonic and biofilm FNV resulted in the lowest superoxide generation (**Figure 2C**). These subspecies also followed the same pattern for overall superoxide release expressed as areas under the curve (AUC; **Supplementary Figure 2C**). The most rapid response was again stimulated by pFNP, whilst the slowest response was induced by pFNV, which also was significantly slower compared to bFNV (**Supplementary Figure 1C**).

### 3.3 NET release by human neutrophils upon *F. nucleatum* stimulation is not subspecies-specific

**Figure 3:**
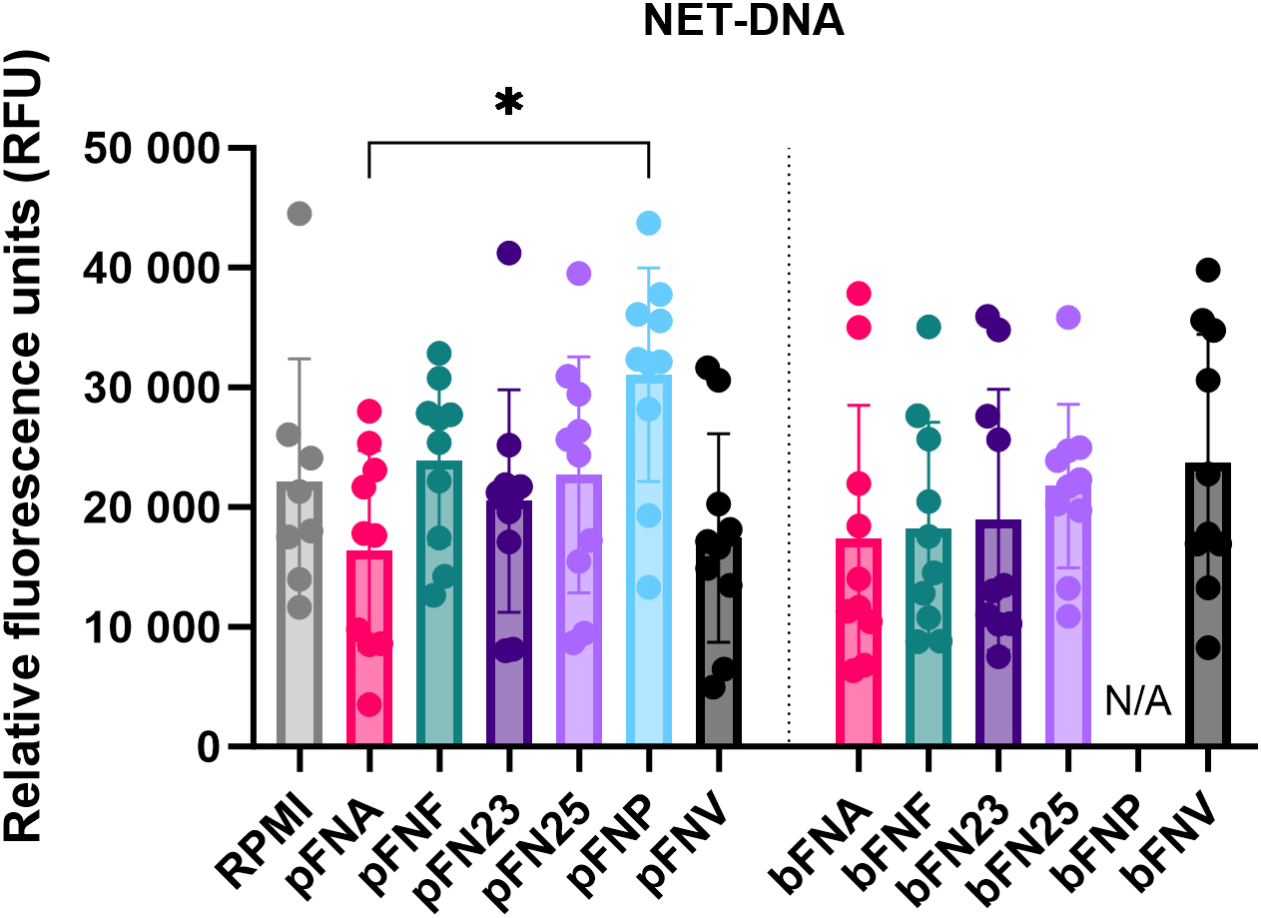
Quantification of extracellular NET-DNA based on the detection of fluorescence signal at 525 nm. Prefix “p” denotes planktonic subspecies, prefix “b” denotes biofilm-grown subspecies. Data were analysed using one-way ANOVA followed by Tukey’s *post hoc* test. Data are shown as mean values ± SD. N=8 for RPMI, N=10 for test samples. *p=0.03.

In addition to quantification of ROS, NET release was quantified and NET formation was confirmed microscopically (**Supplementary Figure 3**). Overall, pFNP stimulated the highest mean amount of NET-DNA (**Figure 3**), however, this was significantly higher compared to pFNA only. A high degree of variation was observed across the subspecies and none of the test samples were significantly different from the negative control (RPMI).

### 3.4 Release of cytokines by human neutrophils in response to *F. nucleatum* is subspecies-specific, whilst release of neutrophil enzymes is subspecies-independent

**Figure 4:**
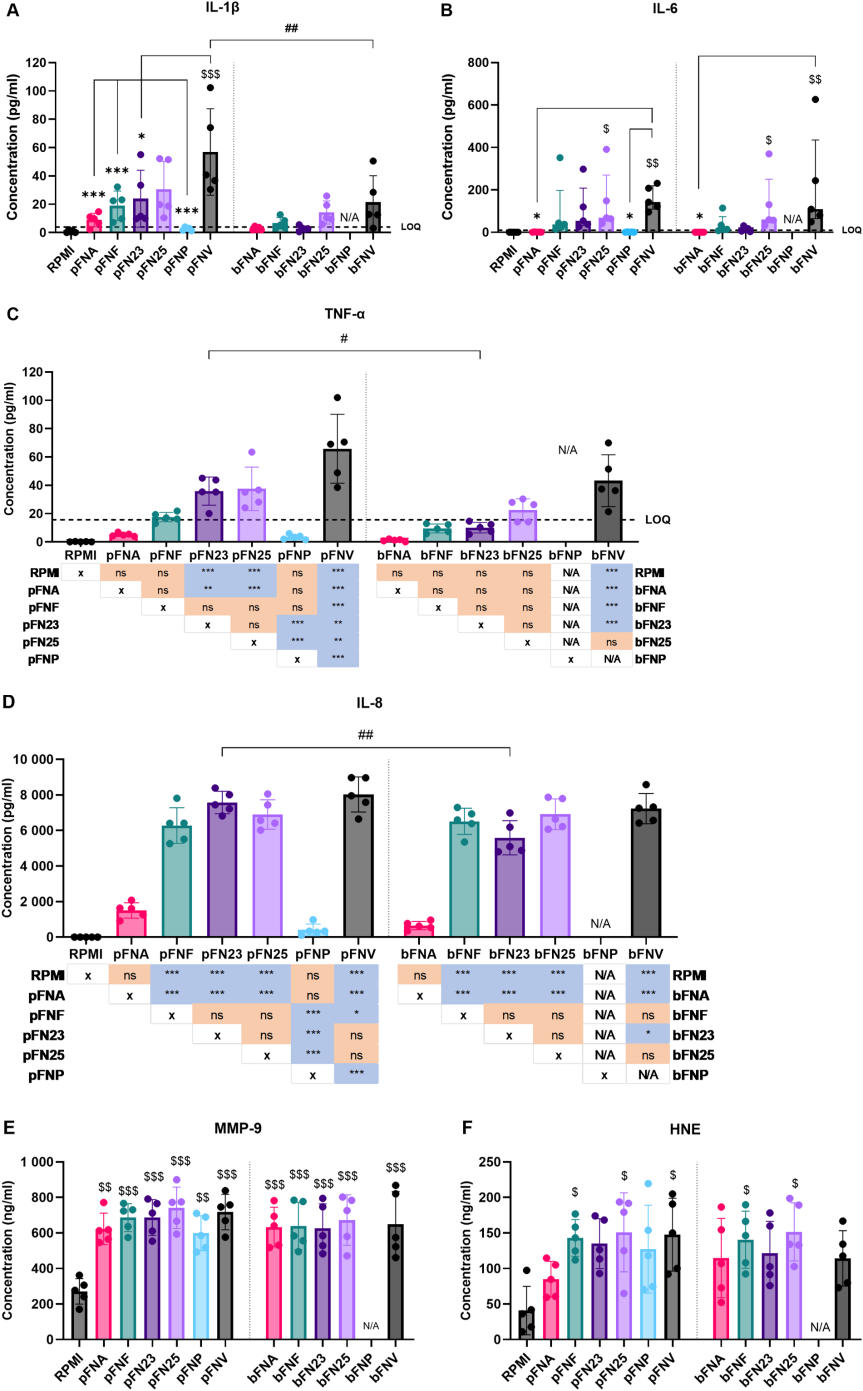
Quantification of cytokine and antimicrobial enzyme release by neutrophils stimulated by *F*. *nucleatum* subspecies. Prefix “p” denotes planktonic subspecies, prefix “b” denotes biofilm-grown subspecies. **(A)** Quantification of IL-1β; LOQ=3.9 pg/ml. **(B)** Quantification of IL-6. Data were not normally distributed and are therefore shown as medians and interquartile ranges. LOQ=9.38 pg/ml. **(C)** Quantification of TNF-α. Statistical significance is detailed in a p-value matrix. LOQ=15.6 pg/ml. **(D)** Quantification of IL-8. Statistical significance is detailed in a p-value matrix. **(E)** Quantification of MMP-9 with no statistical difference among the tested subspecies. **(F)** Quantification of HNE with no statistical difference among the tested subspecies. Subspecies indicated with “$ (dollar)” symbols are significantly higher compared to the negative control (RPMI). Significant differences between planktonic and biofilm-grown subspecies are indicated by “# (hash)” symbols. Significant differences within the groups of planktonic and biofilm-grown subspecies are indicated by “* (asterisk)” symbols. Normally distributed data were analysed using one-way ANOVA followed by Tukey’s *post hoc* test. Non-normally distributed data were analysed using Kruskal-Wallis test followed by Dunn’s *post hoc* test. Assays were performed in technical duplicate, N=5 healthy adult volunteers. Data in A, C, D, E and F are shown as mean values ± SD. LOQ – limit of quantitation. */#/$ - p<0.05; **/##/$$ - p<0.01; ***/$$$ - p<0.001.

Next, neutrophils were incubated with *F. nucleatum* subspecies and the release of selected pro-inflammatory cytokines and neutrophil enzymes after 18 hours of incubation was quantified. Subspecies pFNA, pFNP and bFNA elicited the lowest cytokine release, which in some cases was below the limit of quantitation (LOQ) (**Figures 4A–D**). On the other hand, subspecies pFNV and bFNV triggered the highest mean cytokine release. With respect to neutrophil enzymes MMP-9 and HNE, the release was not stimulated in a subspecies-specific fashion. No significant differences were found among the subspecies and between planktonic and biofilm-grown subspecies (**Figures 4E, F**).

**Figure 5:**
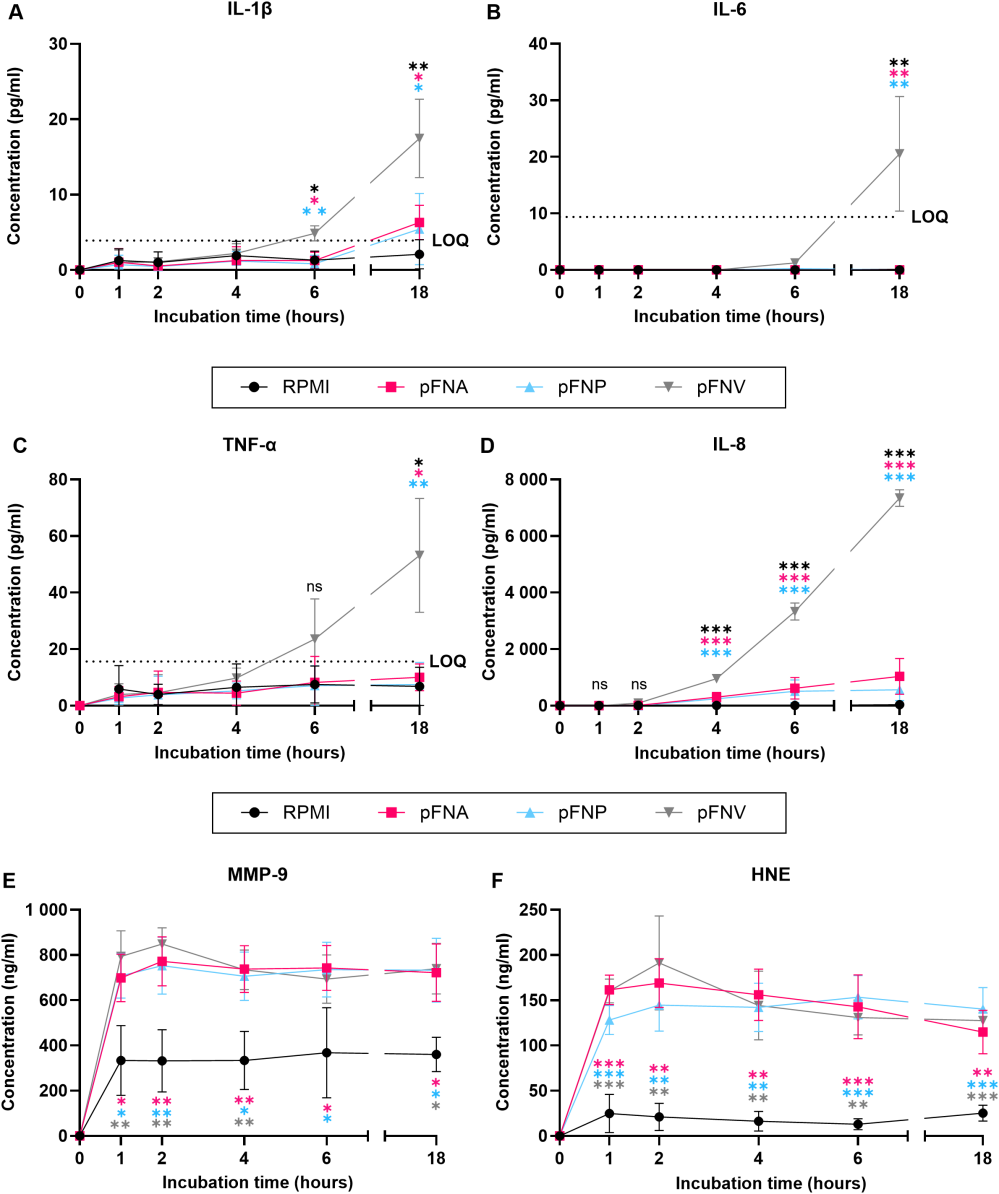
Time-course release of neutrophil cytokines and enzymes over 18 hours stimulated by *F*. *nucleatum* subspecies. Prefix “p” denotes planktonic subspecies. **(A)** Release of IL-1β; LOQ=3.9 pg/ml. **(B)** Release of IL-6. No statistical analysis was performed at time points 0, 1, 2 and 4 because the concentration of IL-6 detected was 0. LOQ=9.38 pg/ml. **(C)** Release of TNF-α. LOQ=15.6 pg/ml. **(D)** Release of IL-8. **(E)** Release of MMP-9. **(F)** Release of HNE. All data are shown as mean values ± SD. Statistical significance was calculated at individual time points using one-way ANOVA followed by Tukey’s *post hoc* test. Statistical significance is not shown if all values within the same time point were below the LOQ. LOQ – limit of quantitation. ns, not significant; *p<0.05; **p<0.01; ***p<0.001. Significant differences are in comparison to subspecies indicated by colour-coding for each time point. N=3 healthy adult volunteers.

Due to the results obtained, degradation of produced cytokines by subspecies FNA and FNP was hypothesised. Additionally, due to the high concentrations of neutrophil enzymes quantified, we were interested in the kinetics of their release. Therefore, a time course release assay of neutrophil cytokines and enzymes was carried out using pFNA, pFNP and pFNV as stimuli. Intriguingly, subspecies FNA and FNV did not appear to degrade the cytokines as their levels remained stable and significantly lower over the incubation period when compared to pFNV (**Figures 5A–D**). A high amount of both neutrophil enzymes MMP-9 and HNE was released as soon as 1 hour after stimulation and remained relatively constant for the duration of the incubation (**Figures 5E, F**). No differences were found among subspecies at any time point.

### 3.5 *F. nucleatum* subspecies stimulate necrosis in human neutrophils

Since the cytokines were not degraded by *F. nucleatum* subspecies and based on the data obtained, it was hypothesised that subspecies FNA and FNP cause premature death of neutrophils and therefore prevent the production of cytokines. Necrosis was selected as a form of cell death with the greatest destructive potential for host tissues caused by the uncontrolled release of cytotoxic neutrophil contents. The start and rate of necrosis were evaluated in order to determine whether FNA and FNP cause earlier onset of necrosis as well as more rapid necrosis. Start of necrosis was calculated from the highest peak of the second derivative of the necrosis curve, while the rate of necrosis was determined from the highest peak of the first derivative of the curve (**Supplementary Figure 4**). Necrotic morphological changes were confirmed microscopically (**Supplementary Figure 5**).

**Figure 6:**
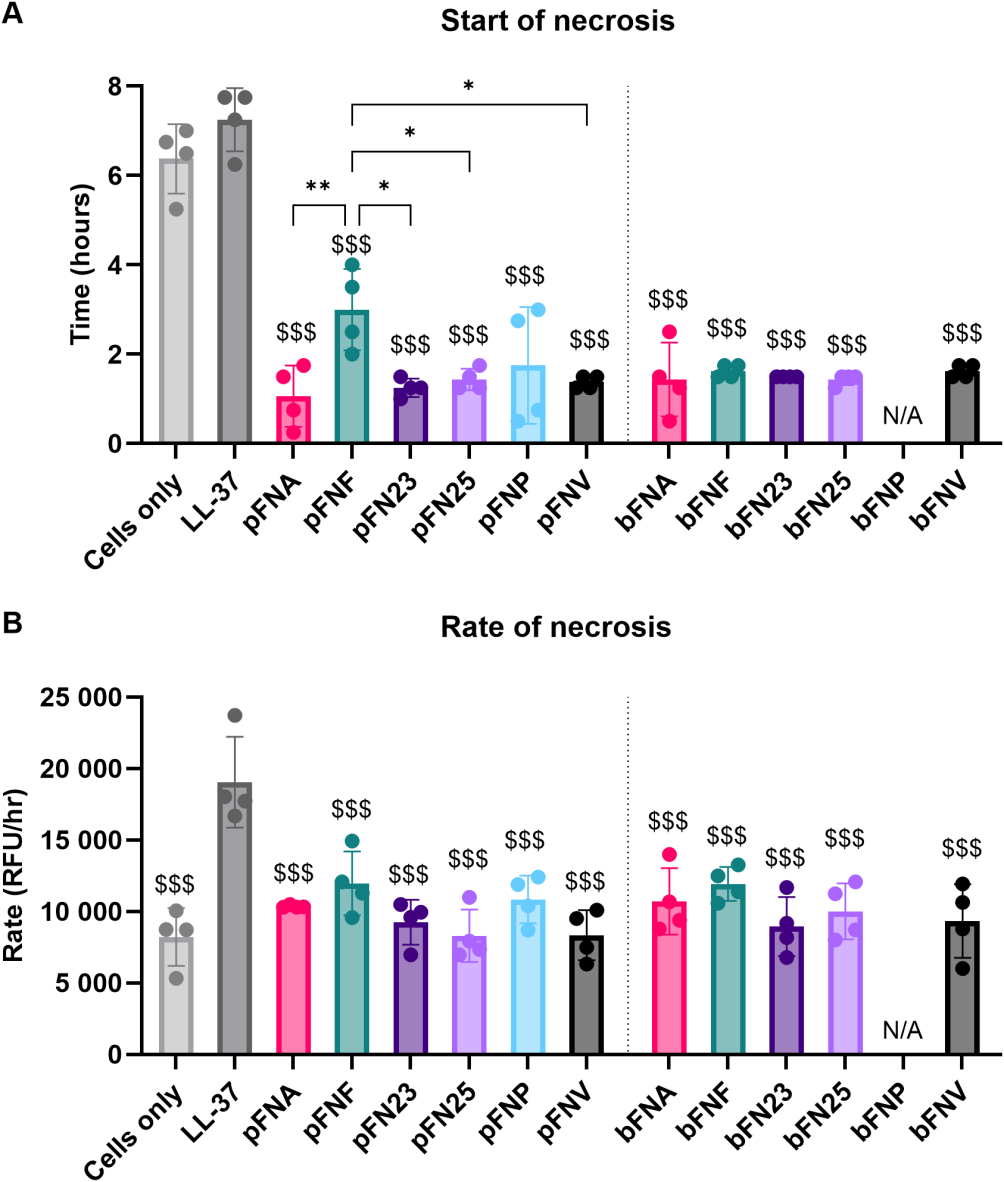
Analysis of neutrophil necrosis triggered by *F. nucleatum* subspecies. Prefix “p” denotes planktonic subspecies, prefix “b” denotes biofilm-grown subspecies. **(A)** Start of necrosis. Test samples were significantly different from the negative control (cells only) and the positive control (LL-37). The level of significance was identical compared to both the negative and the positive control, therefore only one set of symbols is shown using “$ (dollar)” symbols. Significant differences within the groups of planktonic subspecies are indicated by “* (asterisk)” symbols. **(B)** Rate of necrosis. Negative control as well as test samples were significantly different from the positive control LL-37, as indicated by “$ (dollar)” symbols. Assays were performed in technical duplicate, N=4 healthy adult volunteers. All data are shown as mean values ± SD. One-way ANOVA was performed followed by Tukey’s *post hoc* test. *p<0.05; **p<0.01; $$$p<0.001. RFU, relative fluorescence units.

Planktonic FNA, FNP and biofilm-grown FNA, subspecies hypothesised to trigger early necrosis in neutrophils, stimulated the onset of necrosis significantly earlier (1.06, 1.75 and 1.44 hours post stimulation, respectively; **Figure 6**) compared to the necrosis positive control LL-37 (7.25 hours post stimulation). However, this earlier onset of necrosis was seen in all subspecies, whilst significant differences were seen between pFNF and most other planktonic subspecies (**Figure 6A**). Regarding rate of necrosis, all subspecies were significantly lower only when compared to the positive necrosis control LL-37(**Figure 6B**).

## 4 Discussion

Excessive neutrophil responses triggered by *F. nucleatum* have been reported in periodontitis (Matthews et al., 2007; Ling et al., 2015; Chapple et al., 2023) as well as CRC (Kong et al., 2023). In these disorders, *F. nucleatum* resides in biofilms (Chen et al., 2022; Choi et al., 2023), likely expressing biofilm-specific levels of virulence factors (Ali Mohammed et al., 2021) and potentially metabolites, as is the case for another Gram-negative, facultative anaerobe *Salmonella enterica* (Wong et al., 2015). Hence, this study compared for the first time the immunogenicity of biofilm-grown and planktonic *F. nucleatum*. We show that biofilm-grown *F. nucleatum* stimulates significantly higher peak and overall ROS release in the case of total (combining intra- and extracellular ROS) and intracellular ROS. Time to peak total ROS was significantly shorter in response to biofilm-grown stimuli.

Similarly, Hahn and Gunn (2020) measured differential levels of ROS production by neutrophils when comparing planktonic and biofilm-grown *S. enterica* serovars Typhi and Typhimurium. Interestingly, this was serovar-specific, as *S.* Typhi stimulated a higher ROS response in the biofilm state, while *S.* Typhimurium led to higher ROS release in the planktonic state. In addition, the authors systematically tested extracellular polymeric substances (EPS) produced by biofilm forming *Salmonella* and revealed that specific EPS can modulate neutrophil and macrophage responses. Future studies should assess the immunogenicity of EPS components of *F. nucleatum* biofilms in order to distinguish between bacterial cell-mediated and EPS immunogenicity. In our study, biofilms also resulted in significantly lower IL-1β expression. A study by Kaya and colleagues (Kaya et al., 2021) evaluated immune responses of peripheral blood mononuclear cells to planktonic and biofilm *Staphylococcus epidermidis* and quantified significantly lower cytokine release in case of TNF-α, IL-6 and IFN-γ upon biofilm stimulation (Kaya et al., 2021). This, similar to our study, showed that biofilm-grown bacteria elicited lower cytokine production. This could potentially be caused by the biofilm EPS masking recognition sites on the bacterial cells. Alternatively, decreased response could be the result of receptor internalisation due to the presence of multiple stimuli.

Stimulation of neutrophil ROS generation by individual subspecies demonstrated that this process is likely subspecies-specific, with most pronounced differences observed in superoxide generation. Kurgan et al. (2017) also observed subspecies-specificity in a neutrophil-like cell line, however they reported that *F. nucleatum* ssp. *polymorphum* blocked superoxide generation and that ssp. *vincentii* triggered the highest amount of superoxide. This difference could be explained by differences in methodology, as this group used a neutrophil-like cell line (HL-60), while our study analysed the responses of primary human neutrophils. In spite of this discrepancy with our findings, the results show that *F. nucleatum* subspecies significantly differ in their ability to stimulate superoxide generation by neutrophils. Interestingly, Kurgan et al. (2017) also showed *F. nucleatum* subspecies-specific differences in the rate of phagocytosis by HL-60 cells, with the highest phagocytic ingestion in the case of FNP. It is currently unknown whether differences exist in phagocytic clearing of *F. nucleatum* subspecies by human neutrophils, therefore future studies utilising primary neutrophils, as well as planktonically and biofilm-grown bacteria, are warranted.

The importance of superoxide stems from the fact that this specific ROS is the primary radical produced in neutrophils both in the cell and extracellularly. Superoxide radicals can react further in a chain reaction, producing other downstream radical species (Nguyen et al., 2017; Fujii et al., 2022). This is especially critical in the extracellular milieu, where excess of superoxide together with other ROS can cause significant oxidative stress and damage proteins, lipids and other crucial biomolecules (Fujii et al., 2022). Considering that our study demonstrated subspecies-specific production of extracellular superoxide, it can be speculated that individual subspecies have distinct tissue-destructive potential when interacting with neutrophils.

Quantification of NETs revealed that neutrophils released the highest mean amount of NETs upon stimulation with ssp. *polymorphum*. This finding is in agreement with the results of Hirschfeld et al. (2017): the amount of NET-DNA released from neutrophils was higher when stimulated with ssp. *polymorphum* compared to ssp. *nucleatum*, but the difference was not statistically significant, similarly to the current study. Lack of statistical significance in our study was due to considerable inter-donor variation of neutrophil responses. This interindividual variability in various neutrophil responses has frequently been reported elsewhere (Yang et al., 2003; Hirschfeld et al., 2015; Hoffmann et al., 2016), and could be overcome by recruiting more neutrophil donors in future studies.

The release of pro-inflammatory cytokines in our study was highly subspecies-specific, with subspecies *vincentii* consistently stimulating the highest levels of cytokine release and subspecies *animalis* and *polymorphum* the lowest, which was inversely correlated to superoxide release. Similar results were obtained by Ling et al. (2015), who measured cytokine release in neutrophils from healthy volunteers and in patients with periodontitis stimulated with ssp. *polymorphum*, and the concentrations of IL-6, TNF-α and IL-1β, despite statistically significant differences, were markedly low. In combination, these results demonstrate the importance of either careful choice of the *F. nucleatum* subspecies used for experimentation, or of inclusion of more than one *F. nucleatum* subspecies in order to obtain clinically relevant results.

Secretion of neutrophil enzymes in this study was not shown to be subspecies-specific. HNE and MMP-9 are stored in the neutrophil granules, ready to be released rapidly upon bacterial stimulation (Chakrabarti et al., 2006; Zeng et al., 2023). Rapid response was confirmed in this study, as both enzymes were detected in high concentrations as soon as 1 hour post stimulation. Both of these enzymes play a role in the resolution of infection, however their activities have been reported to negatively affect a number of *F. nucleatum*-related diseases. Hiyoshi et al. (2022) demonstrated that HNE exacerbates periodontitis by breaking the gingival epithelial barrier and contributing to periodontal bone loss. In the context of CRC, HNE from NETs was found to significantly increase the migratory activity of CRC cells *in vitro* and inhibition of HNE activity in mice decreased migration of tumour cells to liver tissue, suggesting that HNE plays a role in the formation of liver metastases in CRC (Okamoto et al., 2023).

MMP-9, which can cause pathological connective tissue destruction, is detected in higher amounts in chronic periodontitis (Bildt et al., 2008; Luchian et al., 2022). Higher levels of MMP-9 were quantified in the saliva of patients with earlier stages of periodontitis (localised periodontitis), compared to later stages (generalised periodontitis) (Gursoy et al., 2013). Additionally, increased levels were also measured in gingival crevicular fluid in the early stages of periodontitis (Luchian et al., 2022). Considering that *F. nucleatum* subspecies stimulated a considerably higher release of MMP-9 from neutrophils and that MMP-9 seems to be highly expressed early in periodontitis, future studies could focus on correlations between MMP-9 level and number of *F. nucleatum* ssp. present in periodontal lesions, shedding more light on the role of *F. nucleatum* subspecies in periodontitis development.

Analysis of CRC tumour tissues showed that the expression of MMP-9 is significantly higher compared to the adjacent healthy tissue. Moreover, there was a strong correlation between high MMP-9 expression and pathological parameters such as lymph node and distant metastasis as well as poorer patient survival (Wang et al., 2019). MMP-9 produced by tumours was reported to promote breast cancer progression, metastasis and angiogenesis (Mehner et al., 2014; Guo et al., 2024). Considering that tumour-associated neutrophils were found to be major producers of MMP-9 in tumour tissues (Deryugina et al., 2014) and *F. nucleatum* translocates to breast tissues, colonises them and plays a role in breast cancer metastasis (Guo et al., 2024), the influence of *F. nucleatum*-stimulated MMP-9 release by neutrophils should not be overlooked in future cancer studies.

This study also investigated the potential ability of ssp. *animalis* and *polymorphum* to degrade pro-inflammatory cytokines. Analysis of time-course cytokine release indicated that the selected subspecies did not seem to degrade cytokines released by neutrophils as was initially hypothesised based on the end-point cytokine quantification. Taking together the low cytokine release stimulation with concurrent high superoxide generation in response to ssp. *animalis* and *polymorphum*, it was hypothesised that these subspecies trigger premature disorderly death (necrosis) of neutrophils. This hypothesis was supported by the fact that extracellularly produced superoxide can be pumped back inside the cell, where it can cause oxidative cell damage and potentially cell death if produced excessively (Fujii et al., 2022).

Subsequent analysis of the start of necrosis, however, did not show significant differences in the selected subspecies, except ssp. *animalis*, which stimulated necrosis significantly earlier than ssp. *fusiforme*. Analysis of the rate of necrosis also indicated no differences among the species. Based on the patterns observed, it can be further speculated that these subspecies may modulate cytokine production, potentially at the transcriptional level. Wright et al. (2011) studied transcriptional regulation by ssp. *polymorphum* in human neutrophils and revealed that neutrophils stimulated with this subspecies upregulated genes for cytokines the CXCL-1, CXCL-2 and CXCL-3. However, comparison with the remaining subspecies was not performed, therefore future work should include comparison of individual *F. nucleatum* subspecies and should also include their ability to modulate neutrophil pro-inflammatory gene expression.

Another aspect of *F. nucleatum* subspecies, which may contribute to the pathogenesis of diseases, is the presence of virulence factors. Well-characterised *F. nucleatum* virulence factors include adhesins FplA, Fap2, FadA, RadD and CmpA (Zepeda-Rivera et al., 2024) and a serine protease fusolisin (Bachrach et al., 2004). Nevertheless, the majority of information on their effects comes from CRC studies focusing on their involvement in carcinogenesis. We have previously analysed subspecies-specific differences of adhesins *in silico* (Muchova et al., 2022), yet, the effect of *F. nucleatum* virulence factors has not been investigated in the context of neutrophil antimicrobial responses. While these have not been investigated in the present study, we provide baseline data on the neutrophil responses towards wild-type FN23, the genetically tractable strain. Future studies could draw on these data utilising adhesin- or fusolisin-deficient FN23 strains, thus giving insight into the interaction of *F. nucleatum* virulence factors with human neutrophils.

In an excessive response, release of ROS from neutrophils into the surrounding tissue creates oxidative stress, which leads to tissue damage. Products of host tissue damage serve as nutrients for pathogens resident in this niche, exemplified by the thriving pathogenic community in periodontal pockets (Gasmi Benahmed et al., 2022). Multiple pathogens have developed protective mechanisms, such as antioxidant generation, in response to ROS attack (Li et al., 2021). Based on our results and current literature, it is possible that *F. nucleatum* ssp. *animalis* and *polymorphum* act as more pathogenic subspecies, stimulating higher superoxide release in order to liberate more nutrients during oxidative tissue damage whilst resisting ROS-related killing by neutrophils. Survival of *F. nucleatum* ssp. *nucleatum* and *polymorphum* in human neutrophils was shown by Ellett et al. (2023), which may support this hypothesis.

Considering these two subspecies stimulated very low levels of pro-inflammatory cytokines, they may be able to evade immune clearance by downregulating the expression of neutrophil cytokines, which are important in activating and recruiting additional leukocytes (Fernando et al., 2014; Tecchio et al., 2014; Wajant and Siegmund, 2019). It is conceivable that ssp. *vincentii*, which stimulates very low superoxide and high cytokine production, might be much less virulent, not stimulating excessive oxidative stress and facilitating immune clearance by attracting leukocytes to the site of infection via increased pro-inflammatory cytokine release. In future studies of *F. nucleatum* pathogenicity, it would be interesting to explore whether ssp. *vincentii* and the remaining subspecies survive neutrophil phagocytosis or whether they can be cleared successfully.

We appreciate our *in vitro* study has certain limitations. Firstly, in order to assess immunogenicity of the subspecies, we used single-subspecies biofilms. As *F. nucleatum* is a key bridging organism in biofilms, it usually co-aggregates with many other microorganisms *in vivo* both in healthy (Han, 2015) and diseased states (Chen et al., 2022; Choi et al., 2023). However, we believe it is imperative that the subspecies are first studied individually, without the presence of potentially confounding biofilm-bound bacteria. Future experiments could use multi-species biofilm models to systematically evaluate the impact of individual subspecies on the response of neutrophils to more complex biofilms.

Secondly, our study utilised ATCC strains, which might not accurately reflect the immunogenicity of clinical isolates. Yet, studying commercially available laboratory strains is necessary in order to draw initial generalised conclusions and propose mechanistic hypotheses. Using clinical isolates from patients may shed more light on how specific disease-associated *F. nucleatum* strains interact not only with neutrophils, but also with other host cells.

In summary, the reported study demonstrated that immunogenicity of *F. nucleatum* in human neutrophils is highly subspecies-specific *in vitro* with respect to ROS release and cytokine production, with subspecies FNA and FNP stimulating high superoxide and low cytokine release, while FNV inversely stimulated low superoxide and high cytokine release. This highlights the fact that *F. nucleatum* should not be studied broadly as a species, but rather be considered as individual subspecies as they differ in their capacity to stimulate and likely modulate neutrophil responses. Understanding subspecies-specific immunogenicity of *F. nucleatum* may facilitate the discovery of novel therapeutic targets in *F. nucleatum*-mediated diseases.

## Data Availability

The original contributions presented in the study are included in the article/**Supplementary Material**. Further inquiries can be directed to the corresponding author.
