# Supplementary material for: Fusobacterium nucleatum elicits subspecies-specific responses in human neutrophils
Source: Front Cell Infect Microbiol. 2024 Oct 10;14:1449539. doi: 10.3389/fcimb.2024.1449539 (PMC11499235; doi:10.3389/fcimb.2024.1449539)
Supplement: Supplementary file 1 [file DataSheet1.pdf]

Supplementary Material

Supplementary table 1. Comparison of planktonic (p) and biofilm-grown (b) *F. nucleatum* (FN) stimuli and their immunogenicity in human neutrophils.

Neutrophil response	Entity measured	NC	pFN	bFN	p (NC vs. pFN)	p (NC vs. bFN)	p (pFN vs. bFN)
**Peak release of ROS (RLU)**	Total ROS [Mean (±SD)]	1 394 (646.5) *[N=6]*	29 377 (10 606) *[N=40]*	40 444 (12 971) *[N=40]*	**<0.0001**	**<0.0001**	**0.0001**
	Intracellular ROS [Mean (±SD)]	4 547 (2 783) *[N=6]*	25 547 (9 413) *[N=40]*	34 899 (12 131) *[N=40]*	**<0.0001**	**<0.0001**	**0.0005**
	Superoxide [Median (IQR)]	176.8 (168.3; 195.6) *[N=6]*	1499 (655.5; 3060) *[N=30]*	1848 (1196; 2842) *[N=30]*	**0.0011**	**0.0001**	>0.9999
**Time to peak release of ROS (min)**	Total ROS [Median (IQR)]	-	54.00 (40.00; 66.50) *[N=40]*	45.00 (36.50; 56.00) *[N=40]*	-	-	**0.0439**
	Intracellular ROS [Median (IQR)]	-	48.00 (38.50; 65.00) *[N=40]*	42.00 (34.00; 51.50) *[N=40]*	-	-	0.0521
	Superoxide [Median (IQR)]	-	60.00 (50.00; 68.50) *[N=30]*	53.00 (48.00; 64.00) *[N=30]*	-	-	0.1074
**Overall ROS released (AUC) (RLU x min)**	Total ROS [Mean (±SD)]	163 479 (76 456) *[N=6]*	2 113 547 (528 684) *[N=40]*	2 788 047 (990 395) *[N=40]*	**<0.0001**	**<0.0001**	**0.0005**
	Intracellular ROS [Mean (±SD)]	530 825 (311 930) *[N=6]*	1 848 643 (487 852) *[N=40]*	2 319 971 (924 152) *[N=40]*	**0.0002**	**<0.0001**	**0.0122**
	Superoxide [Median (IQR)]	18452 (17452; 20019) *[N=6]*	132722 (67467; 211290) *[N=30]*	141925 (99069; 204718) *[N=30]*	**0.0011**	**0.0001**	0.9985
**Release of NETs (RFU)**	Extracellular DNA [Mean (±SD)]	22 142 (10 246) *[N=8]*	20 186 (8 818) *[N=50]*	19 999 (9 728) *[N=50]*	0.8471	0.8195	0.9945
**Cytokine release (pg/ml)**	IL-1β [Median (IQR)]	0.3300 (0.000; 1.585) *[N=5]*	19.74 (9.925; 38.76) *[N=25]*	5.270 (2.920; 12.61) *[N=25]*	**<0.0001**	0.0560	**0.0014**

	IL-6 [Median (IQR)]	0.000 (0.000; 0.2650) *[N=5]*	54.36 (19.57; 145.2) *[N=25]*	31.33 (4.660; 94.81) *[N=25]*	**0.0011**	**0.0139**	0.6101
	TNF-α [Median (IQR)]	0.000 (0.000; 0.3050) *[N=5]*	28.13 (14.67; 44.44) *[N=25]*	12.36 (5.590; 27.16) *[N=25]*	**0.0001**	**0.0183**	0.0644
	IL-8 [Median (IQR)]	3.430 (2.010; 6.285) *[N=5]*	6647 (5388; 7883) *[N=25]*	6387 (4841; 7121) *[N=25]*	**0.0003**	**0.0057**	0.4973
**Neutrophil enzyme release (ng/ml)**	MMP-9 [Mean (±SD)]	271.4 (71.69) *[N=5]*	690.9 (98.02) *[N=25]*	644.4 (131.7) *[N=25]*	**<0.0001**	**<0.0001**	0.3229
	HNE [Mean (±SD)]	40.86 (34.13) *[N=5]*	132.3 (44.58) *[N=25]*	128.5 (43.46) *[N=25]*	**0.0002**	**0.0004**	0.9495

No peak occurred in the NC in Time to peak release of ROS, thus only pFN and bFN were compared. Values shown as mean (± standard deviation) or median (interquartile range; IQR) stated in square brackets. Normally distributed data (total and intracellular peak ROS; total and intracellular overall ROS; NET-DNA; neutrophil enzymes) were analysed using one-way ANOVA followed by Tukey's post hoc test. The remaining non-normally distributed datasets were analysed using Kruskal-Wallis test followed by Dunn's post hoc test. Datasets in the section "Time to peak release of ROS" were analysed using Mann-Whitney U test. The number of samples for each dataset is stated in square brackets in italics. NC – negative control. pFN – planktonically-grown *F. nucleatum*, bFN – biofilm-grown *F. nucleatum*; ROS – reactive oxygen species; p – statistical significance value; values in bold highlight significance. RLU – relative light units; AUC – area under the curve; RFU – relative fluorescence units.

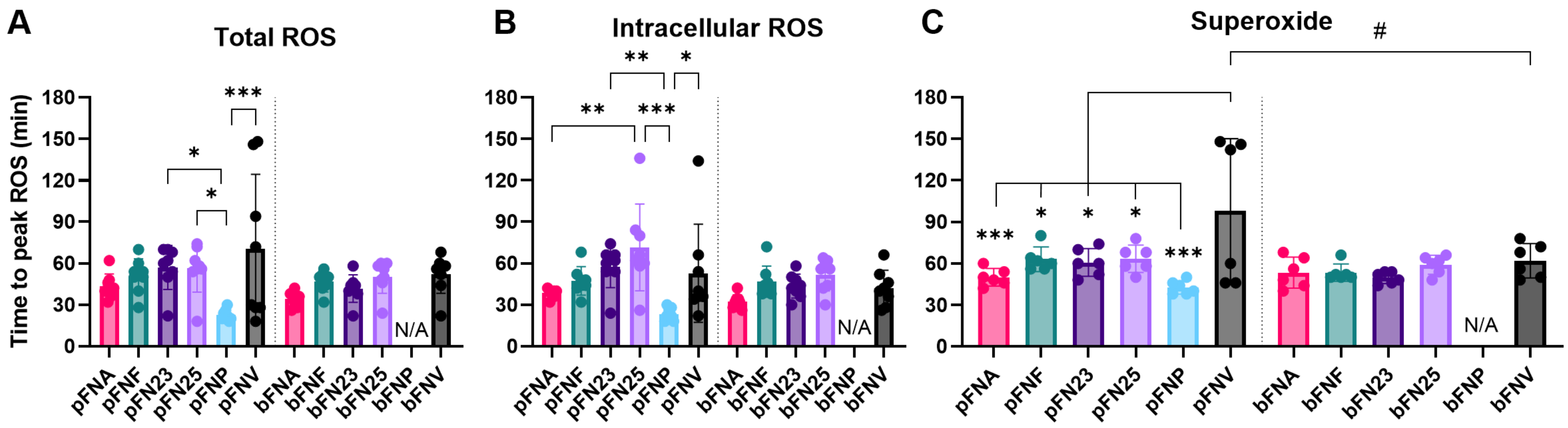

Supplementary figure 1. Time measured to release of peak ROS. (**A**) Total peak ROS. (**B**) Intracellular peak ROS. (**C**) Peak superoxide. pFNV was significantly higher compared to the remaining planktonic subspecies. Biofilm-grown FNP (bFNP) was unavailable due to the absence of biofilm formation. Significant differences between planktonic and biofilm-grown subspecies are indicated by "#" symbols. Statistical significance was calculated using One-way ANOVA with Tukey's post hoc test. */# - p<0.05; ** - p<0.01; *** - p<0.001.

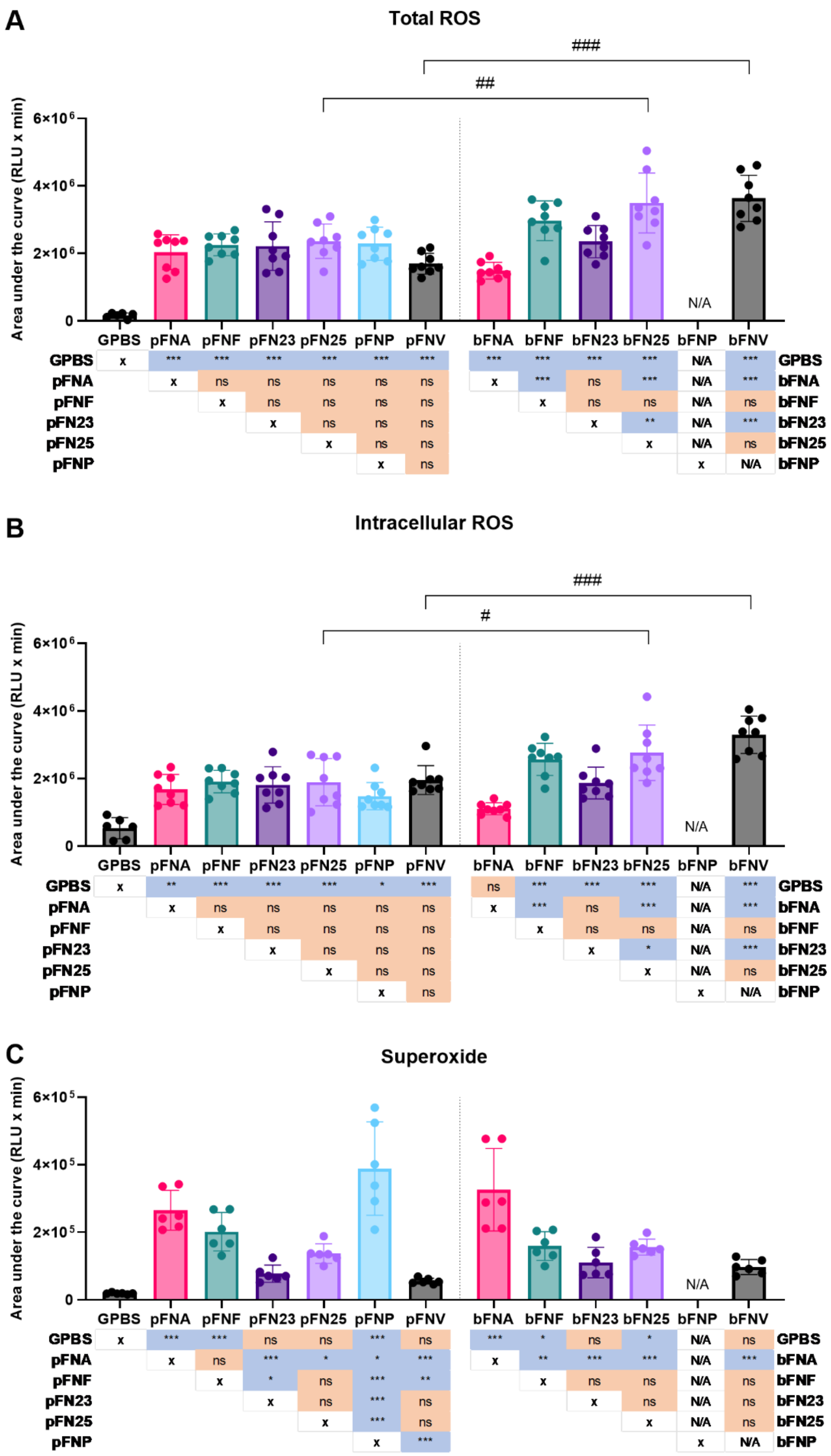

Supplementary figure 2. Overall ROS released expressed as area under the curve. (**A**) Overall total ROS release. N(NC)=6; N(test samples)=8. (**B**) Overall intracellular ROS release. N(NC)=6;

N(test samples)=8. (**C**) Overall superoxide release. N=6. Biofilm-grown FNP (bFNP) was unavailable due to the absence of biofilm formation. Significant differences between planktonic and biofilm-grown subspecies are indicated by "#" symbols. One-way ANOVA was performed followed by Tukey's post hoc test and statistical significance is detailed in p-value matrices. */# - $p<0.05$; **/## - $p<0.01$; ***/### - $p<0.001$.

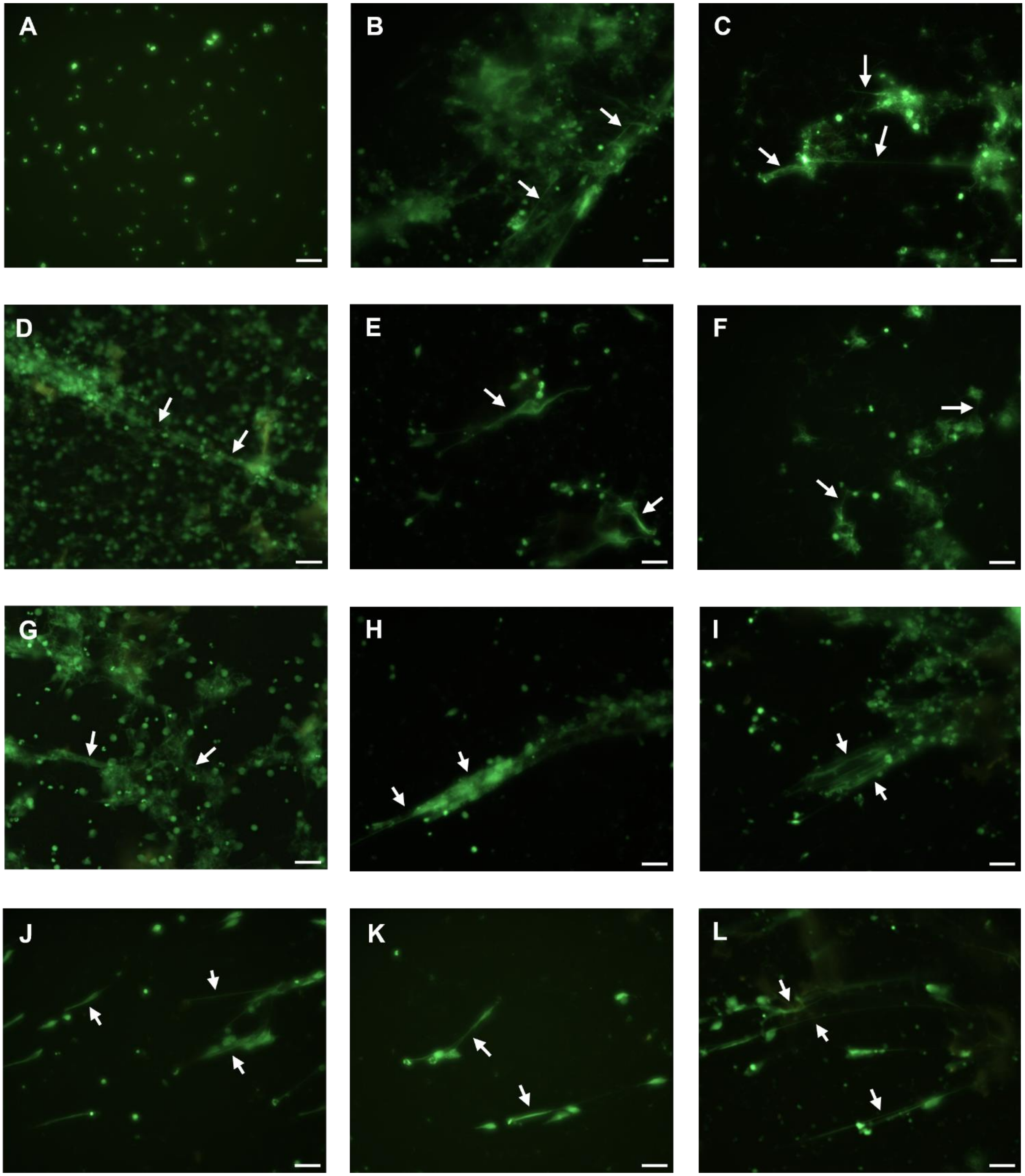

Supplementary figure 3. Fluorescent micrographs showing NETs released by human neutrophils stimulated with planktonic (p) and biofilm-grown (b) *F. nucleatum* (FN) subspecies. **(A)** Unstimulated neutrophils (negative control). **(B)** Neutrophils stimulated with planktonic FNA (pFNA), **(C)** pFNF, **(D)** pFN23, **(E)** pFN25, **(F)** pFNP, **(G)** pFNV, **(H)** biofilm FNA (bFNA), **(I)** bFNF, **(J)** bFN23, **(K)** bFN25, **(L)** bFNV. White arrows point at extruded NET-DNA. All images were taken at 20X magnification. Scale bar: 50 µm.

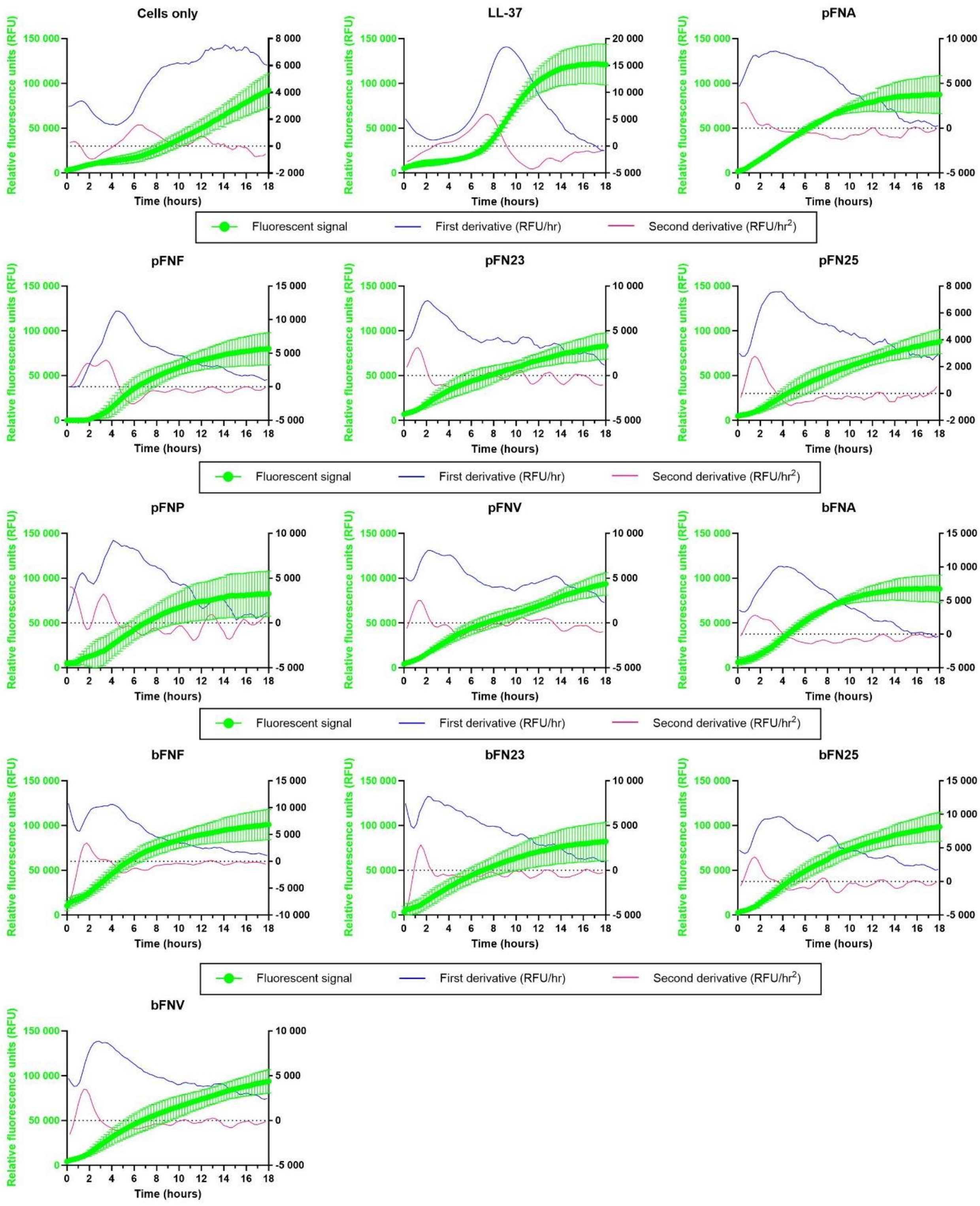

Supplementary figure 4. Necrosis curves with their respective first and second derivative curves. Left y-axis shows the relative fluorescence units (RFU). The right y-axis shows the first and second derivative of the necrosis curves. The highest peak in the first derivative (blue line) indicates the

maximum rate of necrosis while the highest peak in the second derivative (pink line) shows the start of necrosis. Experiments were performed in technical duplicate, N=4 donors.

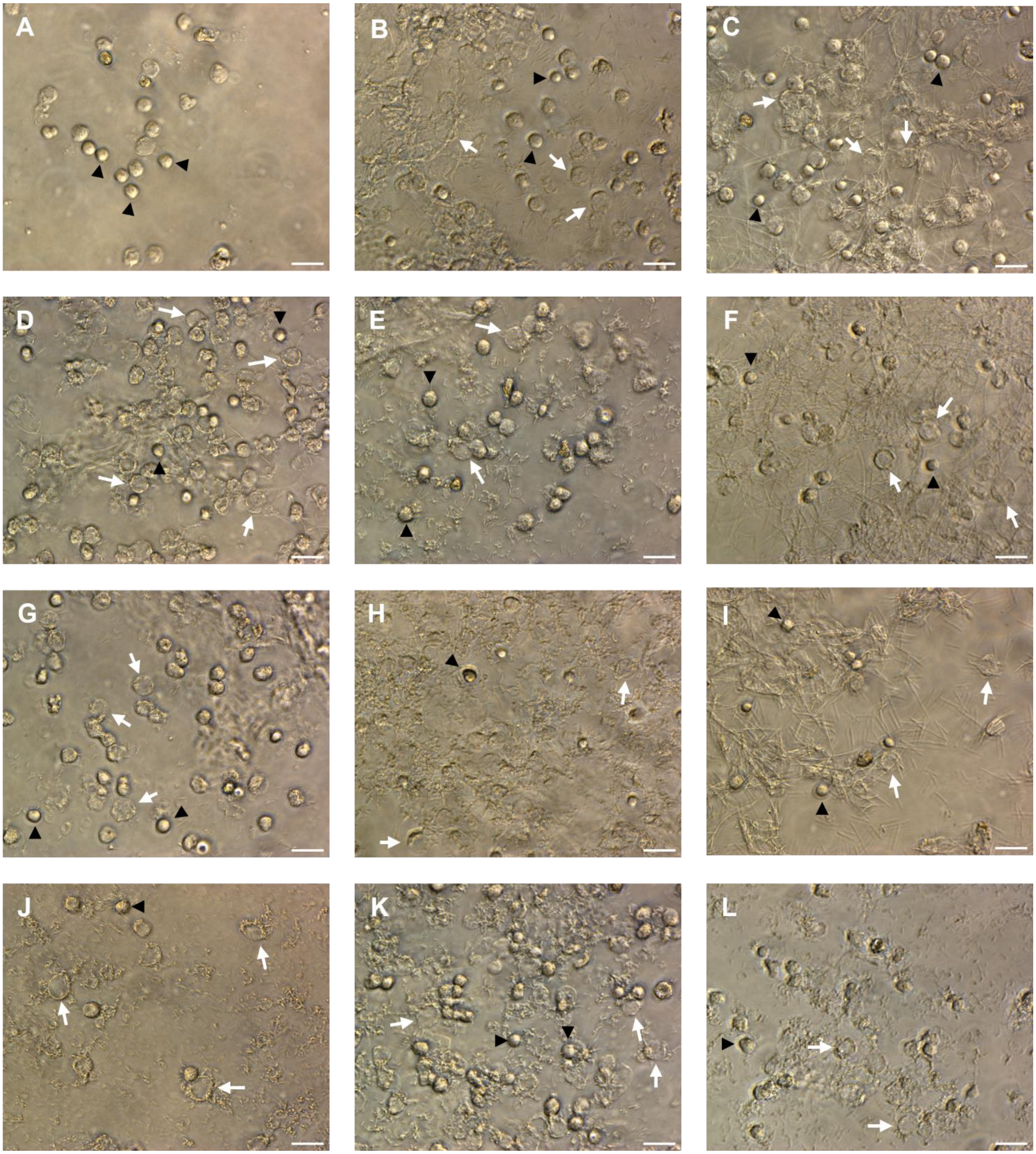

Supplementary figure 5. Light micrographs confirming necrotic morphological changes of neutrophils stimulated with planktonic (p) and biofilm-grown (b) *F. nucleatum* (FN) subspecies. Images were taken after 330 minutes of incubation with bacterial stimuli. **(A)** Unstimulated neutrophils (negative control). **(B)** Neutrophils stimulated with planktonic FNA (pFNA), **(C)** pFNF, **(D)** pFN23, **(E)** pFN25, **(F)** pFNP, **(G)** pFNV, **(H)** biofilm FNA (bFNA), **(I)** bFNF, **(J)** bFN23, **(K)** bFN25, **(L)** bFNV. White arrows: necrotic neutrophils showing ballooning, black arrow heads: neutrophils with normal appearance. All images were taken at 60X magnification. Scale bar: 20 µm.
